# Ambulatory Health Care Visits Among Active Component Members of the U.S. Armed Forces, 2023

**Published:** 2024-06-20

**Authors:** Armed Forces Health Surveillance Division Defense Health Agency, US

## Abstract

**What are the new findings?:**

In 2023 the rate of ambulatory visits in U.S. military and non-military medical facilities was 14.8 visits per person-year, 9.9% lower than the 2022 rate. This decline was primarily driven by a decrease in administrative (ICD-10 Z code) visits. Excluding administrative visits, the crude annual rate of 11.8 visits per person-year for illnesses and injuries in 2023 was approximately 17% higher than the corresponding rates in 2021 and 2019. The numbers and rates of primary causes for ambulatory visits have increased in 14 out of 18 diagnostic categories from 2019 to 2023, except for respiratory system, infectious and parasitic diseases, ‘Other’, and COVID-19. Musculoskeletal, mental, and nervous system or sense organ disorders remain the leading causes of ambulatory visits, with substantial increases from 2019 to 2023. The absolute increase in the number of ambulatory visits for mental health disorders was the highest, with 648,730 total additional visits.

**What is the impact on readiness and force health protection?:**

Disorders of the musculoskeletal, mental, and nervous system and sensory organ major diagnostic categories are already known to have significant impacts on the well-being of military personnel and operational readiness. Unaddressed musculoskeletal injuries and mental health disorders may lead to prolonged periods of unoccupied time, reduced ability to meet the physical and psychological demands of military service, and contribute to attrition.

## 1 BACKGROUND

This report documents the frequencies, rates, trends, and characteristics of ambulatory health care visits in 2023 of active component members of the U.S. Army, Navy, Air Force, Marine Corps, and Space Force. Ambulatory visits of U.S. service members in fixed military and non-military (reimbursed through the Military Health System) hospitals and clinics are documented by standardized records that are routinely archived for health surveillance purposes in the Defense Medical Surveillance System (DMSS). Ambulatory visits not routinely and completely documented within fixed military and non-military hospitals and clinics (e.g., during deployments, field training exercises, or at sea) are not included in this analysis. Additionally, this is the second year in which DMSS data were housed and downloaded for analysis from the MHS (Military Health System) Information Platform (MIP). Although the transition to MIP is complete, data quality assessments of the ICD-10 Z codes for completeness and coding practices, comparing prior and current electronic medical reporting systems, are ongoing. Consequently, data on Z-codes presented in this report are considered provisional but current as of April 18, 2024.

As in prior *MSMR* reports, all records of ambulatory health care visits by active component service members (ACSMs) were categorized according to the International Classification of Diseases, 10th Revision (ICD-10) codes entered in the primary (first-listed) diagnostic position of the visit records. Incidence rates were calculated per 1,000 person-years (p-yrs). Percent change in incidence was calculated using unrounded rates.

**Frequencies, Rates, and Trends**

**Figure 1:**
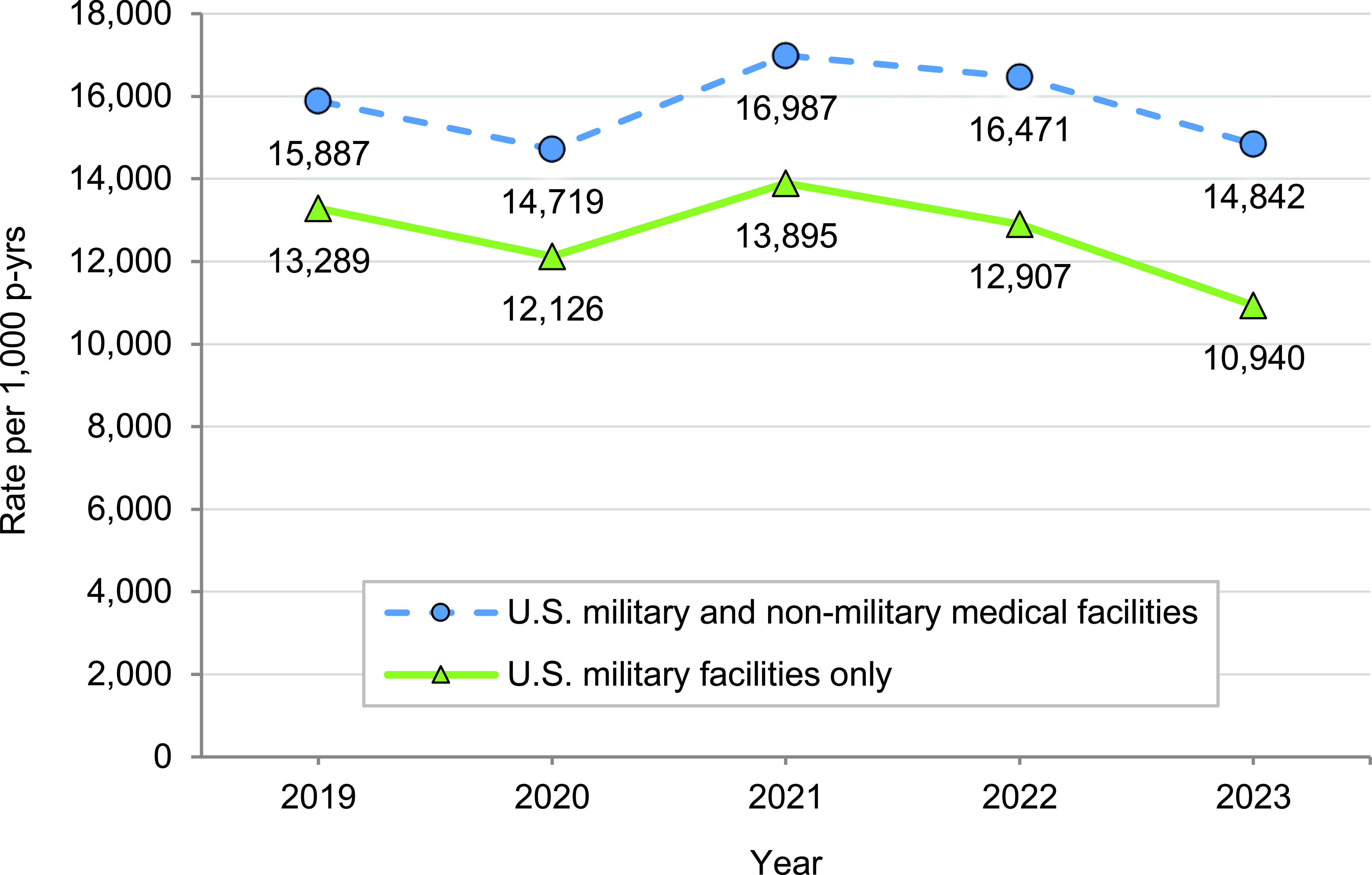
Rates of Ambulatory Visits by Year, Active Component, U.S. Armed Forces, 2019-2023

In 2023, U.S. ACSMs completed 18,882,769 ambulatory visits for medical care, resulting in a crude annual rate (for all causes) of 14,842.0 visits per 1,000 p-yrs or 14.8 visits per p-yr (**Table 1**). The observed rate was the lowest within the current reporting period, declining from its peak in 2021 (**Figure 1**). This decline was driven by a sharp reduction (3,823,905 fewer visits than in 2019, 47.9% rate decrease; 5,246,933 fewer visits than in 2021, 55.4% rate decrease) in the recorded number of administrative (ICD-10 Z code) visits. The ‘Z code’ used in the first diagnostic position identifies the care in the ‘Other’ major diagnostic category (i.e., other factors influencing health status and contact with health services, excluding pregnancy). In contrast to previous years, this year’s reduction resulted in the ‘Other’ category dropping to the second rank among the categories for ambulatory visits in 2023, with musculoskeletal system disorders taking the leading position (**Table 1**).

Z-coded encounters are generally not billable to insurance and are normally used for administrative and other agency-specific requirements. The military uses these Z codes to document some of the burden in the health care system imposed by readiness requirements; examples include routine and special medical examinations, e.g., periodic, occupational, or retirement, along with immunizations, counseling, deployment-related health assessments, suspected exposure to infectious diseases, and screening. From 2019 to 2023, over half of visits (51.6%) attributed to this major diagnostic category included 3 ICD-10 Z codes: encounters for administrative examinations (Z02; n=10,284,975), immunization (Z23; n=4,418,526), and other special examinations without complaint, suspected, or reported diagnosis (Z01; n=3,516,579), which includes examinations for eyes and vision, ears and hearing, blood pressure, dental examination and cleanings, and gynecological exams (data not shown).

The 14,995,126 documented ambulatory visits in 2023 for illnesses and injuries (ICD-10: A00–T88, including relevant pregnancy Z-codes) not including diagnoses classified as ‘Other’ resulted in a crude annual rate of illness- and injury-related visits of approximately 11.8 visits per p-yr, which is approximately 17% higher than the corresponding rates in 2021 (10.1 visits per p-yr) and 2019 (10.0 visits per p-yr).

**Ambulatory Visits, by ICD-10 Major Diagnostic Categories**

Four major diagnostic categories accounted for almost three-quarters (74.8%) of all illness- and injury-related ambulatory visits among ACSMs (not including diagnoses classified as ‘Other’) in 2023: musculoskeletal system/connective tissue disorders (34.6%), mental health disorders (18.3%), disorders of the nervous system and sense organs (11.9%), and signs, symptoms and ill-defined conditions (10.0%) (**Table 1**). Among visits for illness and injury, COVID-19 encounters represented 0.3% of visits in 2023, a substantial decrease from 1.1% of visits in 2021 (data not shown).

In general, the relative distributions of ambulatory visits by ICD-10 diagnostic categories remained stable throughout the surveillance period (**Table 1**). The numbers and rates of ambulatory visits increased in 14 of 18 major diagnostic categories of illness and injury from 2019 to 2023, except for respiratory system, infectious and parasitic diseases, ‘Other’, and COVID-19. Neoplasms, disorders of the nervous system/sense organs, digestive, and circulatory systems had rate increases exceeding 20%. Rate increases surpassed 30% from 2019 to 2023 for major diagnostic categories such as hematologic/immune and mental health disorders, and endocrine, nutrition, and immunity-related conditions. Of note, the absolute increase in the number of ambulatory visits was highest for mental health conditions, totaling additional 648,730 visits (35.3% rate increase), followed by musculoskeletal disorders (498,222 more visits, 14.3% rate increase). Adjustment disorders accounted for the leading diagnosis in this major diagnostic category, for both men and women (**Tables 2** and **3**). Although the increase in the rate of encounters for congenital anomalies exceeded 30%, the absolute change in the frequency of encounters (7,090 more visits) remained the lowest of all major diagnostic categories. While congenital anomalies did not constitute a most frequent diagnosis for women, over a quarter (25.9%) of the congenital anomalies in men were attributed to congenital deformities of feet, including congenital *pes planus* (flat foot) and congenital *pes cavus* (high arch) (**Table 2**). Unspecified and iron deficiency types of anemia were among the leading diagnoses within hematologic and immune disorders major diagnostic category, accounting for 25.7% and 56.4% of diagnoses among service men and women, respectively (Tables 2 and 3).

The largest declines of illness- and injury-specific major diagnostic categories were observed for COVID-19 (-72.8%), infectious and parasitic diseases (-13.2%), and disorders of the respiratory system (-7.8%). Unspecified viral infection and unspecified acute upper respiratory infection were the leading diagnoses in 2023 for infectious and parasitic diseases and disorders of the respiratory system, respectively (**Tables 2** and **3**). Consistent with prior years, diagnostic ‘S codes’ (injury), as opposed to ‘T codes’ (burns and poisonings), accounted for nearly 90% of all ambulatory encounters within this major diagnostic category (data not shown).

**Ambulatory Visits, by Sex**

In 2023, service men accounted for nearly three-fourths (71.4%) of all illness- and injury-related visits, but the annual crude rate among service women (19.1 visits per p-yr) was 87.5% higher than the rate among men (10.2 visits per p-yr) (data not shown). Excluding pregnancy- and delivery-related visits, which accounted for 9.1% of all non-Z-coded ambulatory visits among service women, the illness and injury ambulatory visit rate was 17.4 visits per p-yr, 70.5% higher than the rate among men.

**Figure 2:**
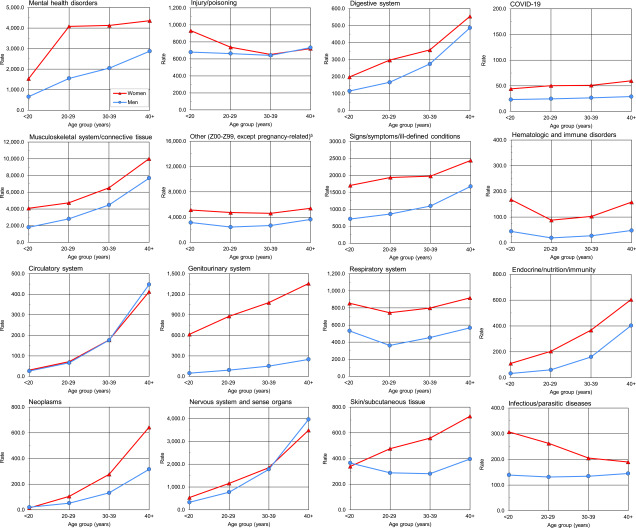
Rates of Ambulatory Visits by ICD-10 Major Diagnostic Category, Age Group, and Sex, Active Component, U.S. Armed Forces, 2023

The female rates of illness- and injury-specific diagnoses exceeded male rates by 50% in all major diagnostic categories, except for diagnoses relating to nervous system and sense organs, circulatory system, digestive system, and injury (data not shown). Female rates were more than twice those of male rates for conditions in hematological, mental, genitourinary, and endocrine-, nutrition- and immunity-related disorder categories. Relationships between age group and ambulatory visit rates were broadly similar among men and women across diagnostic categories (**Figure 2**). Ambulatory rates for neoplasms, disorders in nervous, digestive, circulatory systems, and endocrine-, nutrition- and immunity-related conditions rose more steeply with advancing age than other categories of illness or injury (**Figure 2**).

The 4 leading diagnoses among ambulatory visits were the same for both male and female service members, although the rates for women exceeded those among men: pain in joint (women: 2,239.3; men: 1,514.1; female:male rate ratio [RR]: 1.5); lower back pain (women: 801.8; men: 556.3; RR: 1.4); adjustment disorders (women: 816.3; men: 347.0; RR: 2.4); and pain in the limb, hand, foot, fingers, or toes (female: 444.6; male: 289.4; RR: 1.5) (data not shown). Four other diagnoses were among the 10 most common diagnoses for both men and women: post-traumatic stress disorder (PTSD), cervicalgia (neck pain), unspecified anxiety disorder, and sleep apnea. Sleep apnea was the second-most frequent illness- or injury-specific primary diagnosis during ambulatory visits for men but ranked ninth among women. The difference in the rate rank order of mental disorders is also worth noting. While alcohol dependence was the sixth most frequent diagnosis among men, it was not identified among the 10 leading causes of ambulatory visits for women (**Tables 2** and **3**). Generalized anxiety and major depressive disorders completed the list of 10 most common diagnoses among women.

## 2 DISCUSSION

Ambulatory visits in 2023 among ACSMs declined to the lowest rate observed in the last 5 years. This decline was primarily driven by a decrease in the number and rate of administrative (ICD-10 Z code) ambulatory visits for the ‘Other’ diagnostic category that includes factors influencing health status and contact with health services. As indicated earlier, data quality assessments of Z codes for completeness and coding practices following DMSS data transition to the MIP are ongoing, and consequently Z-code data are provisional. When excluding visits documented by ICD-10 Z-codes, the rate of illness- and injury-specific ambulatory visits were elevated compared to 2019 and 2021. Notably, since 2019 the rate of ambulatory visits for mental health disorders increased by over 35%. The rate of encounters for COVID-19 decreased by nearly 73% from 2021 to 2023. The rate of encounters related to the infectious disease and respiratory system major diagnostic category continued to decline from 2019 to 2023.

While the National Ambulatory Medical Care Survey of 2019 indicates that civilian women use health care services more than men (3.7 vs. 2.7 visits per p-yr, respectively), the sex-specific rate ratio for illness and injury-specific ambulatory encounters indicates a larger disparity among ACSMs (19.1 vs. 10.2 visits per p-yr, respectively).^1^ Furthermore, the crude annual rate of illness- and injury-related visits (11.8 visits per p-yr) among ACSMs far exceed the rate of ambulatory office visits among civilians aged 15-24 years (1.6 visits per p-yr) and 25-44 years (2.0 visits per p-yr).1 Future analyses comparing the major diagnostic category rates to civilian counterparts may be useful to further explicate the costs of readiness.

Several limitations should be considered when interpreting these findings. Ambulatory care at the unit level by non-credentialed providers (e.g., medics, corpsmen) and at deployed medical treatment facilities (including ships at sea) are not included. This summary does not reflect the fact that the nature and rates of illnesses and injuries may vary between deployed and non-deployed ACSMs.

The transition to a new electronic health record for the Military Health System, MHS GENESIS, has introduced new limitations. In previous *MSMR* reports, dispositions following ambulatory visits described a proportion of encounters classified as limited duty, convalescence in quarters, or no limitation. These findings were not included in this as well as prior annual reports, due to a substantial increase in missing disposition data. Disposition information may be included in future reports if data completeness issues can be resolved. Prior reports have described the number of virtual versus in-person ambulatory encounters; however, data quality issues have also been identified regarding the variable delineating this encounter type and is an area of active inquiry.

This summary is based on primary (first-listed) diagnosis codes reported on ambulatory visit records, and the current summary discounts morbidity related to comorbid and complicating conditions that may have been documented in secondary diagnostic positions in health care records. The accuracy of reported diagnoses likely varies according to medical condition, clinical setting, care provider, and treatment facility, as the information is collected for non-surveillance purposes. Although specific diagnoses during individual encounters were potentially not definitive, final, or even correct, summaries of the frequencies, nature, and trends of ambulatory encounters among ACSMs provide descriptive evidence to inform further research and evaluation.

Rates and frequencies reported herein do not reflect unique individuals, but a rate of total ambulatory visits per person-year. This report documents all ambulatory health care visits but does not estimate incidence rates for the diagnoses described. These data provide descriptors for health care provision, which elevate rates for disorders requiring increased numbers of ambulatory visits. In contrast to common, self-limited, and minor illnesses and injuries that require very little, if any, follow-up or continuing care, illnesses and injuries necessitating multiple ambulatory visits for evaluation, treatment, and rehabilitation are over-represented in this summary.

## Figures and Tables

**Table 1:** Numbers, Rates^a^, and Ranks^b^ of Ambulatory Visits, by ICD-10 Major Diagnostic Category, Active Component, U.S. Armed Forces, 2019, 2021 and 2023

	2019			2021			2023		
Major Diagnostic Category (ICD-10)	No.	Rate	Rank	No.	Rate	Rank	No.	Rate	Rank
Musculoskeletal system (M00-M99)	4,693,803	3,569.3	2	4,476,003	3,354.4	2	5,192,025	4,080.9	1
Other (Z00–Z99, except pregnancy-related)^c^	7,711,548	5,864.1	1	9,134,576	6,845.7	1	3,887,643	3,055.7	2
Mental disorders (F01-F99)	2,101,124	1,597.8	3	2,419,888	1,813.5	3	2,749,854	2,161.4	3
Nervous system and sense organs (G00-G99, H00-H95)	1,483,388	1,128.0	4	1,554,969	1,165.3	4	1,777,326	1,396.9	4
Signs, symptoms, and ill-defined conditions (R00-R99)	1,314,084	999.3	5	1,471,785	1,102.9	5	1,498,649	1,177.9	5
Injury and poisoning (S00-T88, DOD0101-DOD0105)	846,667	643.8	6	784,027	587.6	6	859,792	675.8	6
Respiratory system (J00-J99, U07.0)	690,950	525.4	7	491,067	368.0	7	616,311	484.4	7
Skin and subcutaneous tissue (L00-L99)	406,876	309.4	8	414,320	310.5	9	431,295	339.0	8
Pregnancy and delivery (O00-O9A, relevant Z codes)^d^	380,998	1,720.5	9	419,198	1,813.3	8	389,880	1,737.8	9
Genitourinary system (N00-N99)	303,079	230.5	10	333,691	250.1	10	343,274	269.8	10
Digestive system (K00-K95)	252,836	192.3	11	274,849	205.9	11	312,837	245.9	11
Infectious and parasitic diseases (A00-B99)	232,145	176.5	12	216,476	162.2	12	195,013	153.3	12
Endocrine, nutrition, immunity (E00-E89)	145,931	110.9	13	162,298	121.6	13	191,502	150.5	13
Circulatory system (I00-I99)	140,517	106.9	14	159,288	119.4	14	170,999	134.4	14
Neoplasms (C00-D49)	127,436	96.9	15	137,065	102.7	16	148,925	117.1	15
Hematologic and immune disorders (D50-D89)	38,937	29.6	16	45,795	34.3	17	50,232	39.5	16
COVID-19 (U07.1, U09.9)	0	0	0	147,383	110.5	15	38,260	30.1	17
Congenital anomalies (Q00-Q99)	21,862	16.6	17	23,443	17.6	18	28,952	22.8	18
Total, illness and injury-specific ambulatory visits	13,180,633	10,023.0		13,531,545	10,140.9		14,995,126	11,786.2	
Total	20,892,181	15,887.1		22,666,121	16,986.6		18,882,769	14,841.9	

Abbreviations: ICD-10, International Classification of Diseases, 10th Revision; No., number; COVID-19, coronavirus disease 2019.

^a^ Rate per 1,000 person-years.

^b^ Rank of major diagnostic category based on number of ambulatory visits.

^c^ Other factors influencing health status and contact with health services (excluding pregnancy-related).

^d^ Rate of pregnancy and delivery-related hospitalizations among females only.

**Table 2:** Numbers and Percentages of the Most Frequent Diagnoses During Ambulatory Visits Among Men by ICD-10 Major Diagnostic Category, Active Component, U.S. Armed Forces, 2023

Diagnostic category (ICD-10 codes)	No.	%^a^
**Infectious and parasitic diseases (A00–B99)**	**140,491**	
Viral infection, unspecified	19,257	13.7
Viral wart, unspecified	9,298	6.6
Tinea unguium	8,000	5.7
Viral intestinal infection, unspecified	7,696	5.5
Plantar wart	6,728	4.8
**Neoplasms (C00–D49)**	**105,661**	
Neoplasm of uncertain behavior of skin	10,826	10.2
Melanocytic nevi, unspecified	8,006	7.6
Melanocytic nevi of trunk	4,386	4.2
Benign lipomatous neoplasm, unspecified	3,609	3.4
Malignant neoplasm of testis, unspecified whether descended or undescended	3,079	2.9
**Endocrine, nutrition, immunity (E00–E89)**	**129,255**	
Hyperlipidemia, unspecified	15,904	12.3
Testicular hypofunction	13,789	10.7
Vitamin D deficiency, unspecified	12,377	9.6
Obesity, unspecified	12,118	9.4
Type 2 diabetes mellitus without complications	10,101	7.8
**Hematologic and immune disorders (D50–D89)**	**26,996**	
Anemia, unspecified	4,572	16.9
Other specified disorders of white blood cells	3,082	11.4
Sickle-cell trait	2,570	9.5
Iron deficiency anemia, unspecified	2,376	8.8
Glucose-6-phosphate dehydrogenase (G6PD)	1,769	6.6
**Mental health disorders (F01-F99)**	**1,865,164**	
Adjustment disorders	363,609	19.5
Alcohol dependence	251,997	13.5
Post-traumatic stress disorder (PTSD)	244,356	13.1
Anxiety disorder, unspecified	124,357	6.7
Generalized anxiety disorder	100,989	5.4
**Nervous system and sense organs (G00–G99, H00–H95)**	**1,438,567**	
Sleep apnea	640,137	44.5
Myopia	98,240	6.8
Chronic pain, not elsewhere classified	60,391	4.2
Insomnia	47,963	3.3
Astigmatism	34,275	2.4
**Circulatory system (I00–I99)**	**142,371**	
Essential (primary) hypertension	68,436	48.1
Scrotal varices	4,774	3.4
Atherosclerotic heart disease of native coronary artery	3,084	2.2
Acute embolism and thrombosis of deep veins of lower extremity	2,824	2.0
Pulmonary embolism without acute cor pulmonale	2,741	1.9
**Respiratory system (J00-J99, U07.0)**	**440,823**	
Acute upper respiratory infection, unspecified	107,129	24.3
Acute pharyngitis, unspecified	41,849	9.5
Allergic rhinitis due to pollen	38,151	8.7
Allergic rhinitis, unspecified	26,726	6.1
Acute nasopharyngitis [common cold]	22,958	5.2
**Digestive system (K00–K95)**	**239,103**	
Gastroesophageal reflux disease without esophagitis	30,672	12.8
Noninfective gastroenteritis and colitis, unspecified	21,534	9.0
Constipation	9,814	4.1
Hemorrhage of anus and rectum	9,418	3.9
Melena	9,114	3.8
**Genitourinary system (N00–N99)**	**128,471**	
Other specified disorders of male genital organs	25,484	19.8
Male erectile dysfunction, unspecified	14,559	11.3
Calculus of kidney	8,624	6.7
Hypertrophy of breast	6,814	5.3
Male infertility, unspecified	4,875	3.8
**Skin and subcutaneous tissue (L00–L99)**	**316,496**	
Pseudofolliculitis barbae	44,269	14.0
Acne vulgaris	23,085	7.3
Dermatitis, unspecified	21,153	6.7
Ingrowing nail	17,272	5.5
Pilonidal cyst and sinus without abscess	9,189	2.9
**Musculoskeletal system and connective tissue (M00–M99)**	**3,926,429**	
Pain in joint	1,586,638	40.4
Low back pain	582,899	14.8
Pain in limb, hand, foot, fingers, and toes	303,247	7.7
Cervicalgia	181,424	4.6
Dorsalgia, unspecified	121,230	3.1
**Congenital anomalies (Q00–Q99)**	**20,820**	
Congenital pes planus	2,558	12.3
Other specified congenital malformations of skin	2,197	10.6
Congenital pes cavus	1,709	8.2
Other congenital deformities of feet	1,131	5.4
Atrial septal defect	1,043	5.0
**Symptoms, signs and abnormal clinical and laboratory findings, NEC (R00–R99)**	**1,055,320**	
Other symptoms and signs involving emotional state	69,932	6.6
Headache, unspecified	54,936	5.2
Chest pain, unspecified	53,999	5.1
Other abnormalities of breathing	42,504	4.0
Unspecified abdominal pain	37,493	3.6
**Injury/poisoning (S00–T98, DOD0101–DOD0105)**	**696,955**	
Sprain of ankle	40,985	5.9
Sprain of shoulder joint	29,489	4.2
Concussion	26,778	3.8
Sprain of cruciate ligament of knee	26,669	3.8
Tear of meniscus, current injury	17,077	2.5
**Other (Z00–Z99, except pregnancy-related)^b^**	**2,814,185**	
Encounter for immunization	304,634	10.8
Encounter for other administrative examinations	299,303	10.6
Encounter for administrative examinations, unspecified	273,263	9.7
Other specified counseling	172,099	6.1
Encounter for other specified special examinations	122,413	4.3

Abbreviations: ICD-10, International Classification of Diseases, 10th Revision; No., number; G6PD, glucose-6-phosphate dehydrogenase; NEC, not elsewhere classified.

^a^ Percentage of the total number of ambulatory visits within the diagnostic category.

^b^ Other factors influencing health status and contact with health services (excluding pregnancy-related).

^c^ Z-code data are provisional but current as of April 18, 2024.

**Table 3:** Numbers and Percentages of the Most Frequent Diagnoses During Ambulatory Visits Among Women by ICD-10 Major Diagnostic Category, Active Component, U.S. Armed Forces, 2023

Diagnostic category (ICD-10 codes)	No.	%^a^
**Infectious and parasitic diseases (A00–B99)**	**54,522**	
Viral infection, unspecified	7,961	14.6
Candidiasis of vulva and vagina	5,286	9.7
Viral intestinal infection, unspecified	2,746	5.0
Herpes viral infection, unspecified	2,541	4.7
Other viral agents as the cause of disease classified elsewhere	2,451	4.5
**Neoplasms (C00–D49)**	**43,264**	
Leiomyoma of uterus, unspecified	5,445	12.6
Malignant neoplasm of breast of unspecified site	3,799	8.8
Neoplasm of uncertain behavior of skin	3,279	7.6
Melanocytic nevi, unspecified	2,982	6.9
Benign neoplasm of pituitary gland	1,338	3.1
**Endocrine, nutrition, immunity (E00–E89)**	**62,247**	
Obesity, unspecified	9,052	14.5
Vitamin D deficiency, unspecified	6,762	10.9
Polycystic ovarian syndrome	6,142	9.9
Hypothyroidism, unspecified	6,110	9.8
Overweight	3,000	4.8
**Hematologic and immune disorders (D50–D89)**	**23,236**	
Iron deficiency anemia, unspecified	6,822	29.4
Anemia, unspecified	6,272	27.0
Sickle cell trait	1,413	6.1
Iron deficiency anemia secondary to blood loss (chronic)	1,178	5.1
Other specified disorders of white blood cells	990	4.3
**Mental health disorders (F01-F99)**	**884,690**	
Adjustment disorders	183,137	20.7
Post-traumatic stress disorder (PTSD)	137,021	15.5
Anxiety disorder, unspecified	74,244	8.4
Generalized anxiety disorder	72,271	8.2
Major depressive disorder, recurrent, moderate	49,478	5.6
**Nervous system and sense organs (G00–G99, H00–H95)**	**338,759**	
Sleep apnea	55,667	16.4
Myopia	37,234	11.0
Chronic pain, not elsewhere classified	22,712	6.7
Migraine, unspecified	18,470	5.5
Insomnia	15,061	4.4
**Circulatory system (I00–I99)**	**28,628**	
Essential (primary) hypertension	10,601	37.0
Varicose veins of lower extremities with other complications	1,067	3.7
Supraventricular tachycardia	926	3.2
Venous insufficiency (chronic) (peripheral)	870	3.0
Raynaud's syndrome	843	2.9
**Respiratory system (J00-J99, U07.0)**	**175,488**	
Acute upper respiratory infection, unspecified	45,547	26.0
Acute pharyngitis, unspecified	19,291	11.0
Allergic rhinitis due to pollen	15,320	8.7
Allergic rhinitis, unspecified	11,032	6.3
Acute nasopharyngitis [common cold]	10,019	5.7
**Digestive system (K00–K95)**	**73,734**	
Constipation	10,930	14.8
Gastroesophageal reflux disease without esophagitis	8,069	10.9
Noninfective gastroenteritis and colitis, unspecified	7,543	10.2
Unspecified hemorrhoids	2,389	3.2
Hemorrhage of anus and rectum	2,278	3.1
**Genitourinary system (N00–N99)**	**214,803**	
Acute vaginitis	18,170	8.5
Urinary tract infection, site not specified	16,261	7.6
Stress incontinence (female) (male)	15,597	7.3
Abnormal uterine and vaginal bleeding, unspecified	14,313	6.7
Other specified noninflammatory disorders of vagina	12,291	5.7
**Pregnancy and delivery (O00-O99, relevant Z codes)**	**389,880**	
Encounter for care and examination of lactating mother	47,869	12.3
Pregnant state, incidental	31,985	8.2
Encounter for supervision of normal first pregnancy	20,205	5.2
Encounter for supervision of other normal pregnancy	17,491	4.5
Encounter for routine postpartum follow-up[	15,162	3.9
**Skin and subcutaneous tissue (L00–L99)**	**114,799**	
Acne vulgaris	19,289	16.8
Dermatitis, unspecified	8,654	7.5
Urticaria, unspecified	3,861	3.4
Ingrowing nail	3,342	2.9
Nonscarring hair loss, unspecified	3,048	2.7
**Musculoskeletal system and connective tissue(M00–M99)**	**1,265,596**	
Pain in joint	502,378	39.7
Low back pain	179,874	14.2
Pain in limb, hand, foot, fingers, and toes	99,750	7.9
Cervicalgia	72,192	5.7
Dorsalgia, unspecified	41,463	3.3
**Symptoms, signs and abnormal clinical and laboratory findings, NEC (R00–R99)**	**443,329**	
Pelvic and perineal pain	30,711	6.9
Headache, unspecified	28,448	6.4
Unspecified abdominal pain	24,839	5.6
Other symptoms and signs involving emotional state	24,290	5.5
Chest pain, unspecified	14,911	3.4
**Injury/poisoning (S00–T98, DOD0101–DOD0105)**	**162,837**	
Sprain of ankle	12,900	7.9
Concussion	7,343	4.5
Sprain of cruciate ligament of knee	6,841	4.2
Unspecified injury of ankle and foot	4,209	2.6
Injury of muscle, fascia and tendon of abdomen, lower back and pelvis	3,710	2.3
**Other (Z00–Z99, except pregnancy-related)^b^**	**1,073,458**	
Encounter for other administrative examinations	103,127	9.6
Encounter for administrative examinations, unspecified	86,826	8.1
Other specified counseling	73,325	6.8
Encounter for immunization	72,721	6.8
Encounter for other specified special examinations	35,352	3.3

Abbreviations: ICD-10, International Classification of Diseases, 10th Revision; No., number; G6PD, glucose-6-phosphate dehydrogenase; NEC, not elsewhere classified.

^a^ Percentage of the total number of ambulatory visits within the diagnostic category.

^b^ Other factors influencing health status and contact with health services (excluding pregnancy-related).

^c^ Z-code data are provisional but current as of April 18, 2024.
